# Neural Radiance Field‐Based 3D Reconstruction and View Synthesis for Mussel Farm Environments

**DOI:** 10.1002/snz2.70044

**Published:** 2026-04-14

**Authors:** Junhong Zhao, Bing Xue, Ross Vennell, Mengjie Zhang

**Affiliations:** ^1^ Centre for Data Science and Artificial Intelligence & School of Engineering and Computer Science Victoria University of Wellington Wellington New Zealand; ^2^ Coastal and Freshwater Group Cawthron Institute Nelson New Zealand

**Keywords:** 3D reconstruction, aquaculture, large‐scale scene reconstruction, mussel farm intelligence, neural radiance field (NeRF)

## Abstract

As the mussel farming industry grows, the demand for advanced monitoring and management solutions intensifies. Traditional methods rely heavily on on‐site observations and frequent boat trips to monitor buoy flotation and other operational elements, often resulting in limited and sporadic assessments that can hinder the decision‐making process. This article proposes using 3D reconstruction techniques to reconstruct mussel farm scenes from the vessel‐captured video footage, allowing for holistic visualizations from various perspectives and enabling comprehensive post analysis of the mussel farm dynamics. While previous efforts relied on traditional Structure from Motion and multiview techniques for mussel farm scene reconstruction, they often struggled to capture fine details and faced challenges with reflective water surfaces due to their reliance on local features. In this work, we are the first to explore and extend the capabilities of neural radiance field (NeRF) for mussel farm reconstruction. To overcome the practical challenges of this unique environment, we propose a multi‐NeRF framework with region‐specific modeling, enabling the capture of both the global scene and finer details of key elements such as buoys. Furthermore, we introduce a geometry regularization method to improve the planar reconstruction of the water surface. Our results demonstrate significant advancements in 3D reconstruction quality over previous methods, particularly in mesh completeness and the precise handling of specular and diffuse texture details while also enabling realistic novel view synthesis. These advancements, designed particularly for mussel farm applications, can contribute to its intelligent monitoring and management by providing a comprehensive understanding of the farm's geometry and dynamics, ultimately facilitating more informed decision‐making processes.

## 1 Introduction

Over the past few years, the aquaculture sector has experienced significant growth globally, with mussel farming playing a key role by supplying premium seafood to both local and international markets. As the industry expands, the demand for efficient management and monitoring solutions for mussel farms has become increasingly vital (Ferreira et al. 2012; Chary et al. 2022).

**FIGURE 1:**
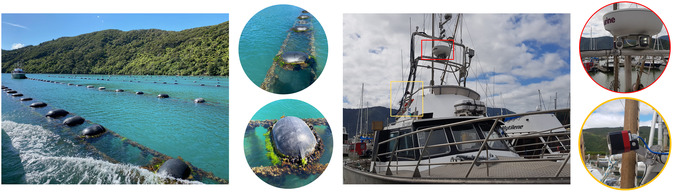
This visualization illustrates the setup of a mussel farm cultivation platform and a marine camera system mounted on vessels. The left side displays the arrangement of buoys, from which ropes with growing mussels are suspended. The right side shows a vessel equipped with marine cameras that capture videos of the mussel farm, along with other data (e.g., GPS) during the vessel's navigation.

Mussel farming involves cultivating mussels by suspending them in the water column on ropes or longlines, where they grow and filter nutrients from the surrounding water. These ropes are anchored by floating buoys, which ensure their stability and position (see Figure 1, left side). Once the mussel spat (young mussels) are collected from natural sources or hatcheries, they are attached to underwater ropes, where they grow and develop over a period of 12–18 months. Throughout this period, the arrangement of buoys and ropes forms the essential framework of the mussel farm, providing crucial support and stability for the cultivation process. One of the main tasks for mussel farmers is to continuously monitor this framework, paying close attention to the condition of the buoys. This is crucial for preventing issues such as sinking or drifting, which can disrupt the underwater structures and lead to potential losses during mussel growth.

Traditional mussel farm monitoring methods often involve labor‐intensive, on‐site procedures such as frequent boat trips to nearshore or open‐ocean farm areas. These methods are costly and provide only limited, spotty assessments (Aquaculture I. 2023). In response to these challenges, 3D reconstruction technology has emerged as a promising solution. Modern vessels are often equipped with marine cameras that can capture detailed scenes on the water surface, including buoy distributions and other operational details during navigation (see Figure 1, right). Leveraging this data can reduce the strain on mussel farmers and enhance on‐site evaluations with comprehensive offsite analysis. By utilizing recorded footage, 3D reconstruction creates precise, reproducible 3D models of the scene (Zhao et al. 2023). This approach allows for the projection of extensive scenes into a compact format while preserving geometric accuracy, enabling holistic visualization and exploration from various perspectives.

**FIGURE 2:**
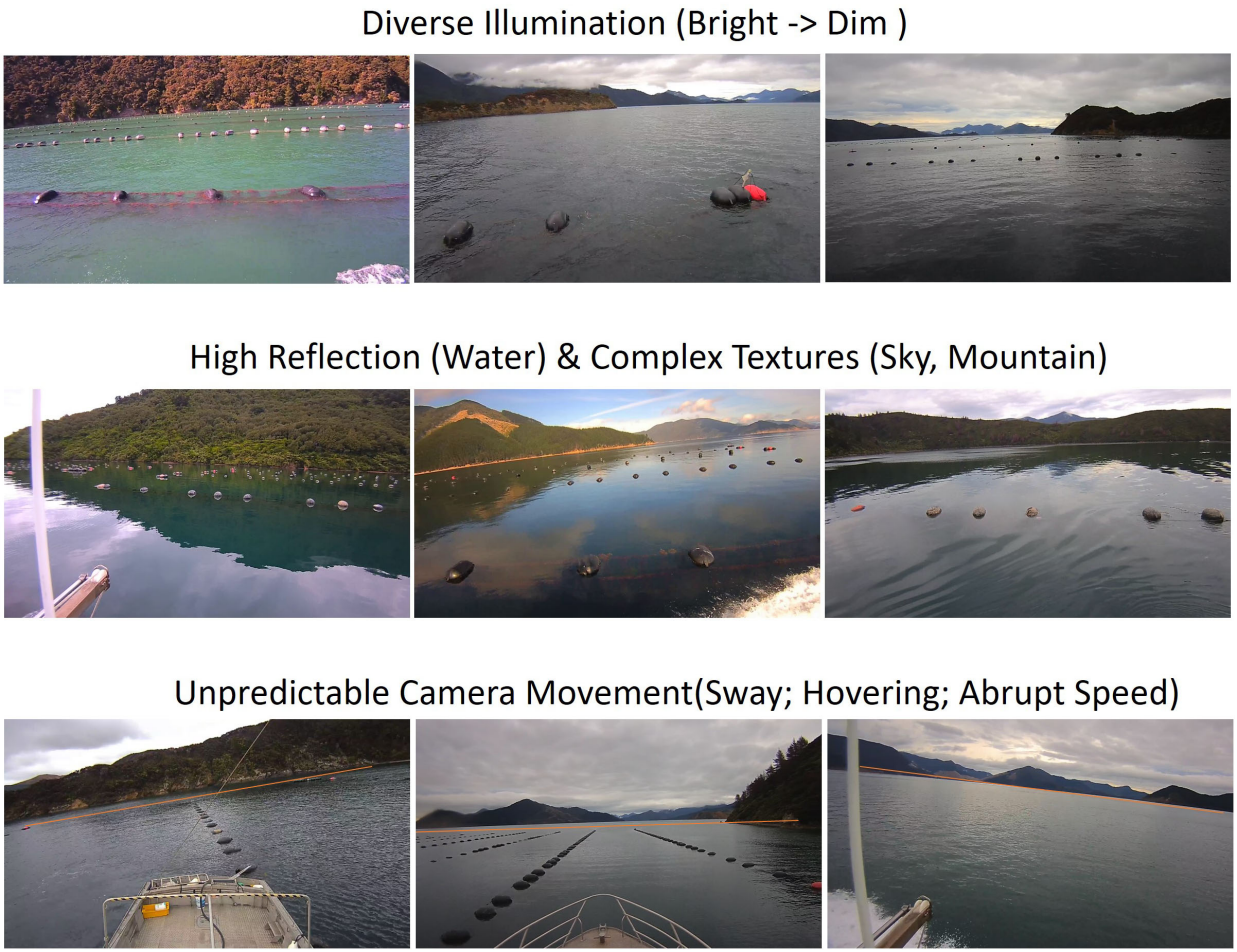
Challenges of 3D reconstruction in mussel farm scenes. The data were captured during the vessel's extended journey, resulting in unconstrained recording with diverse illumination conditions (first row), pronounced reflections and shadows on the water surface (middle row), and unpredictable vessel movements, including oscillation and abrupt speed changes (last row). The buoy, a crucial element for reconstruction, is significantly smaller compared to its background.

However, accurately reconstructing an expansive marine scene poses significant challenges. The data on reflective water surfaces, intricate coastal terrains, and the camera's unpredictable movements introduce difficulties to existing 3D reconstruction methods (see Figure 2). The seminal work by Zhao et al. (2024) and Zhao et al. (2023) employed the Structure from Motion (SfM) method, specifically the COLMAP method, which is a general‐purpose revised version of basic SfM proposed by‐Schonberger and Frahm (2016) to reconstruct a mussel farm. However, SfM techniques rely on local features to match corresponding points/pixels through video frames. These features are often greatly affected by reflections on water surfaces, leading to incomplete reconstructed meshes. Moreover, due to the sparse point cloud representation of the scene, the SfM method often results in coarse and low‐resolution reconstructions with insufficient visual fidelity.

In addition to the background scene, such as mountains and the water surface providing context, the primary focus of mussel farm monitoring is on the distribution and condition of the buoys, which are significantly smaller in scale. A key challenge, therefore, is balancing the large‐scale nature of the scene with the need to preserve fine‐grained details of the buoys during reconstruction. To address this, Zhao et al. (2024) proposed a triangulation method for buoy objects to enhance their fine‐detailed visualization in mussel farm environments. However, this method depends on a predefined 3D object template for the buoys, which can be cumbersome and often results in coarse scaling.

In contrast, we introduce an innovative method based on the neural radiance field (NeRF) for mussel farm reconstruction, specifically designed to enhance the accuracy and completeness of the reconstructed mussel farm 3D models (Mildenhall et al. 2021). NeRF‐based method has sparked a surge of development in recent years, revolutionizing the field of 3D reconstruction with its continuous volumetric representation and neural rendering capabilities (Barron et al. 2022; Zhang et al. 2020; Park et al. 2021; Reiser et al. 2023; Cao et al. 2023; Attal et al. 2023; Martin‐Brualla et al. 2021). It excels at capturing dense geometric and texture details and synthesizes novel views from arbitrary viewpoints with high visual fidelity. In this work, we proposed a novel multi‐NeRF framework to increase its detail levels in both geometry and textural reconstruction. Given the challenging and reflective nature of water surfaces, we also propose a water surface geometry regularization method to improve the reconstruction of the water plane. Furthermore, we decompose its appearance into diffuse and specular components to more effectively handle illumination effects. Our proposed approach not only builds a dense representation of the global scene but also recovers precise buoy instances without assuming any prior geometry information.

In summary, our contributions are as follows:

- We propose a novel NeRF‐based approach to reconstructing large‐scale mussel farm scenes and realizing arbitrary new view synthesis. A pipeline is designed to effectively reconstruct both the overall scene geometry and texture, as well as the specific details at the buoy instance level.
- We propose using multiple NeRF networks to address the diverse complexities of different scene components. By leveraging masks from segmented regions and employing an adaptive ray sampling method, we enable more effective training of NeRF models at varying levels of granularity.
- To improve the reconstruction of the water surface, which poses a significant challenge, we introduce a novel expectation–maximization (EM) algorithm for water plane regularization. This algorithm effectively prompts the reconstructed 3D points of the water area to align with the primary water plane and penalizes outliers that deviate significantly from it during model training.

## 2 Related Work

### 2.1 3D Reconstruction and NeRF Advancements

Traditional 3D reconstruction techniques such as Multi‐view Stereo (MVS) (Schönberger et al. 2016; Seitz et al. 2006) and SfM (Özyeşil et al. 2017; Szeliski 2022) have provided foundational methods for reconstructing 3D scenes. They have seen advancements in scalability and robustness for large‐scale reconstructions (Wu 2011; Cui et al. 2023; Chen et al. 2023). Techniques such as DeepSFM (Wei et al. 2020) and NeuralRecon (Sun et al. 2021) further integrate deep learning to improve feature extraction and matching. In mussel farm reconstruction, the seminal work by Zhao et al. (2023) utilizes the widely adopted COLMAP method (Schonberger and Frahm 2016) for reconstructing mussel farm scene. While this approach provides feasible results, challenges such as oversampled mesh topologies and limited scene detail persist in these traditional methods.

NeRF (Mildenhall et al. 2021) has revolutionized 3D reconstruction by employing volumetric representation through neural networks, delivering higher accuracy and completeness. It has spurred a wave in recent years (Barron et al. 2021; Barron et al. 2022; Zhang et al. 2020; Xiang et al. 2021; Reiser et al. 2021; Park et al. 2021; Reiser et al. 2023; Cao et al. 2023; Attal et al. 2023; Martin‐Brualla et al. 2021). Some efforts have been taken to enhance NeRF's capabilities in large‐scale (indoor/outdoor/city/arial) scenes. For example, Block‐NeRF (Tancik et al. 2022) and Mega‐NeRF (Turki et al. 2022) use a divide‐and‐conquer strategy to reconstruct small blocks in parallel. Block‐NeRF focuses on fixing appearance inconsistencies between blocks, while Mega‐NeRF aims to encourage network sparsity in aerial scenes. Mip‐NeRF (Barron et al. 2021) pioneers the use of Gaussian functions for cone sampling approximation and scale‐aware positional encoding to mitigate aliasing problems. Mip‐NeRF 360 (Barron et al. 2022) further advances Mip‐NeRF by utilizing space contraction to accurately model unbounded scenes. Urban‐NeRF integrates lidar points to supervise depth estimation in outdoor scenes (Rematas et al. 2022), while Switch‐NeRF utilizes a switch transformer to dynamically assign rays to different blocks during training (Zhenxing and Xu 2022).

Although NeRF has demonstrated high rendering quality, its implicit 3D representations pose challenges for surface mesh reconstruction and texture generation. In our mussel farm reconstruction task, we prioritize explicit mesh generation for representing surface topology due to its compatibility with various visualization tools and its scalability in handling detailed and complex scenes, where implicit methods often face limitations or reduced effectiveness.

### 2.2 Mesh and Textures Generation from NeRF

Some research efforts focus on surface reconstruction, where scenes are represented by radiance fields or alternative methods such as the signed distance function (SDF) (Darmon et al. 2022; Fu et al. 2022; Wang et al. 2021; Wu et al. 2022; Yang et al. 2022; Yariv et al. 2021) and unsigned distance fields (UDF) (Ueda et al. 2022; Long et al. 2023). Based on radiance field representation, NeRF2Mesh (Tang et al. 2023) aims to convert NeRF reconstructions into textured meshes by utilizing density fields to accurately capture geometric topology while simultaneously refining appearance to produce both the mesh and texture. It employs the Marching Cubes (Lorensen and Cline 1998) algorithm for surface mesh extraction. This approach is highly adaptable, making it particularly suitable for applications in mussel farm environments. In contrast, SDF‐based methods, such as NeuS (Wang et al. 2021 ) and BakedSDF (Yariv et al. 2023), utilize SDF for transforming densities into meshes, aiming for real‐time rendering. However, these methods often produce overly smoothed geometries and have difficulties with thin structures. Other approaches, such as those investigating UDF or combinations of density fields and SDF (Ueda et al. 2022; Long et al. 2023), address some of these limitations but are typically restricted to object‐level reconstruction. Consequently, these methods are not well suit for the expansive, open‐scale scenarios found in mussel farm environments.

In this work, we utilize NeRF2Mesh (Tang et al. 2023) as our backbone framework to convert radiance fields into detailed meshes and textures. While NeRF2Mesh effectively reconstructs coarse backgrounds, our focus extends to improving water surface reconstruction and capturing fine‐grained elements, such as buoys, to address specific needs in mussel farming.

### 2.3 Water/Fluid Surface Reconstruction

Reconstructing water surfaces (Li et al. 2012; Pickup et al. 2010; Thapa et al. 2020) or dynamic fluids is a complex task. The water surface is unlike commonly studied objects like human faces, bodies, or trees. It lacks visually distinctive features and always exhibits a constantly changing topology, which poses challenges for tracking and matching algorithms. Moreover, obtaining accurate ground truth data in water environments is a challenge, as advanced systems like laser scanners struggle with complex reflection and refraction conditions.

Previous attempts to reconstruct water surfaces often relied on physical constraints such as refraction (Morris and Kutulakos 2011) and reflection (Wang et al. 2009), requiring specialized experimental setups. To overcome these limitations, Li et al. (2012) use viewport videos and a two‐step process involving shape‐from‐shading and shallow water flow estimation. However, this method suffers from low input resolution and still needs the camera to be very close to the water surface. Recently, researchers have used deep neural networks for reconstructing refractive surfaces. For example, Zhan et al. (2023) developed NeRFfrac for synthesizing novel views of water surfaces. This approach trains an MLP‐based Refractive Field to estimate distances from the camera to the refractive surface but requires an orthographic camera positioned above the fluid.

Existing methods often necessitate specific experimental setups or are limited to low‐resolution inputs. In contrast, our data are acquired under unconstrained conditions, comprising single‐viewport videos recorded during navigation in water environments (refer to Figure 2). Conventional methods cannot be applied to this unique data type. Thus, we turn to NeRF techniques, particularly enhancing their efficacy in handling water surfaces. Our objective is to precisely reconstruct both sea surfaces, mountain terrain, and intricate instances of interest from footage captured using an unconstrained, moving camera.

### 2.4 Instance‐Level 3D Reconstruction

In a mussel farm environment, buoys are crucial but typically small and sparse compared to other background elements. Traditional 3D reconstruction techniques, such as MVS and SfM, primarily focus on entire scenes and struggle to reach the precision required for small or intricate objects due to limitations in feature‐matching capabilities. Several studies have sought to improve precision and detail in 3D reconstruction by leveraging deep learning. For instance, COTR (Jiang et al. 2021) explores cotraining strategies for enhanced precision, while PixelNeRF (Yu et al. 2021) leverages neural networks to boost the fidelity of 3D reconstructions, showcasing improvements in capturing detailed information. Despite these advancements, these methods still struggle with data sparsity, movement variations, and object distance, often resulting in suboptimal object‐level reconstruction performance. Zhao et al. (2024a) employ buoy detection and tracking to identify buoy instances first (McMillan et al. 2024, 2025, 2026) and triangulate their 3D positions for reconstruction. Although this method improves reconstruction details, it depends on a predefined 3D buoy template, which can add complexity and lead to inaccurate scaling.

In contrast, our proposed method reconstructs the buoys in an end‐to‐end way without relying on any predefined shapes, allowing for capturing their actual forms and sizes for more precise scaling and detail. We introduce an adaptive ray sampling technique that balances breadth for large‐scale reconstruction and density for instance‐level detail. This approach ensures effective reconstruction of small objects while maintaining computational efficiency.

## 3 Proposed Method

**FIGURE 3:**
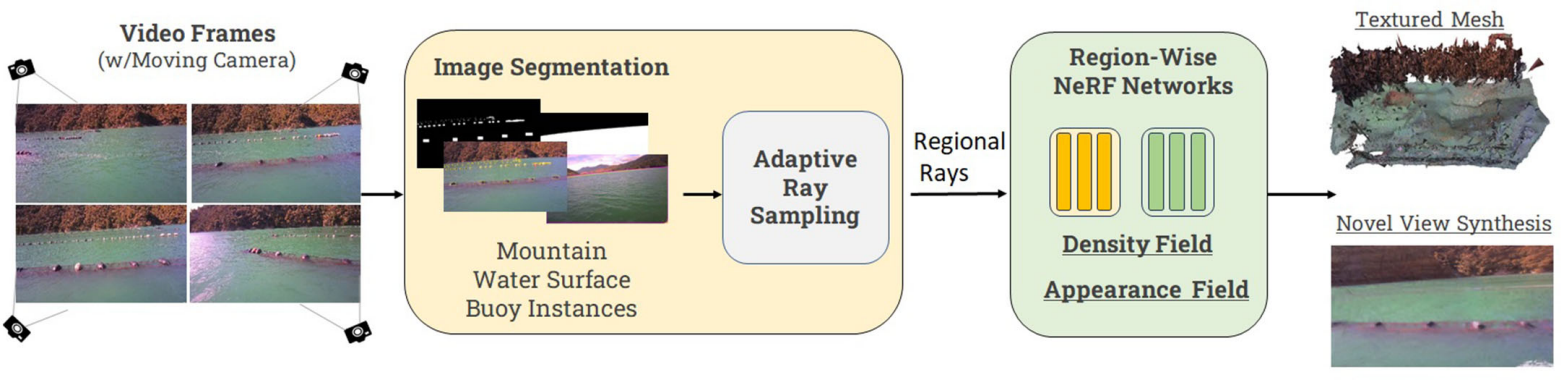
Overall pipeline of our NeRF‐based mussel farm reconstruction. Details on the region‐wise NeRF networks are in Figure 4.

As illustrated in Figure 3, our proposed pipeline begins by detecting the waterline and buoys, effectively separating the background regions, such as the mountains and water, from the buoy regions. The resulting masks guide the construction of distinct regions. A NeRF framework, composed of multiple NeRF networks, is then used to build the density field, with each network dedicated to a specific region of the scene. To effectively train the buoy NeRF, an adaptive ray sampling strategies are applied to this network to ensure enhanced details in the small‐scale reconstruction. After the density fields are established, they undergo a NeRF2Mesh transformation, generating and refining meshes while simultaneously recovering textures. Finally, all components are integrated to produce a cohesive reconstruction of the scene. In the meantime, new viewport images can be synthesized for any desired perspective visualization.

### 3.1 Image Region Segmentation

We perform the waterline detection to separate the scene image into the water surface and the mountain area to reconstruct their 3D structure separately. We adopt the waterline detection method proposed by Zeng et al. (2023), a traditional image processing approach that initially estimates the waterline using edge detection, followed by further refinement through color analysis.

We then develop a buoy detection model to extract bounding boxes for each buoy and distinguish them from the background. To achieve this, we fine‐tuned a YOLOv7 model using an annotated mussel farm image dataset (McMillan et al. 2023), which includes 727 images captured from both boat‐mounted and buoy‐mounted cameras. The model's weights and biases are initialized using a pretrained model from the Microsoft COCO dataset, and all convolutional layers remain trainable during the fine‐tuning process.

### 3.2 Adaptive Ray Sampling

Achieving different levels of detail within the NeRF framework relies heavily on the ray sampling strategy. For large backgrounds, a less dense, uniform sampling approach can capture key features and the overall scene structure. However, for instance‐level recovery, particularly for smaller objects like buoys, the sampling density must be significantly increased to ensure no details are missed. This focus is necessary to allow for higher precision without incurring excessive memory costs. To balance these needs, we propose an adaptive ray sampling method that efficiently captures varying details across regions.

**FIGURE 4:**
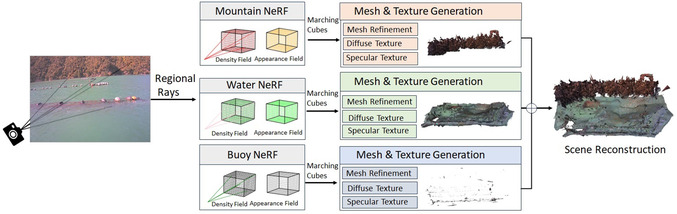
The Region‐wise NeRF network comprises three distinct NeRF models, each focused on specific regions of the scene: mountains, water surface, and buoy instances. Rays are sampled adaptively under the guidance of area‐specific masks, ensuring targeted training for each model. This approach allows for region‐specific refinements, such as improving the visibility of individual buoys and enhancing the details of the water surface. Meshes and textures for each region are generated using cube marching and appearance modeling. These components are then seamlessly integrated to produce a unified 3D reconstruction, enabling novel view synthesis beyond the training dataset.

According to the splitting of the image with the detected waterline and buoy detection, the masks for the water area (denoted as $M_{w}$), mountain background (denoted as $M_{b}$), and instance area (denoted as $M_{i}$) is generated and guided the ray sampling separately (see Figure 4). Let R represent the set of rays cast from the camera into the scene. The ray sampling for the background region (including the mountain and water areas) being under a uniform distribution U(0,|R|) can be formulated as

(1) $$R_{\text{bg}}=\{r\in R|(r\cap M_{w}\;\neq \;\emptyset \;\text{or}\;r\cap M_{b}\;\neq \;\emptyset )\;\text{and}\;r∼U(0,|R|)\}$$

Similarly, the ray sampling for the buoy area can be expressed as

(2) $$R_{\text{buoy}}=\{r\in R|r\cap M_{i}\;\neq \;\emptyset \;\;\text{and}\;r∼U(0,|R|)\}$$

Combining the adaptive sampling strategy with the 3D points sampling, the sampled points along a ray r can be formulated as

(3) $$x_{i}(r)=o+t_{n}d+i\cdot \delta_{i}\frac{d}{\|d{\|}^{2}},$$

where $t_{n}$ represents the near bound ( $t_{n}=0.05$ in our experiments). $\delta_{i}=t_{i+1}-t_{i}$ is the step size between points i and i+1. d is the direction of a ray and i ranges from 1 to N(r)

Thus, the adaptive sampling function $x_{i}$ along ray r can be represented as:

(4) $$x_{i}=\left\{ \begin{array}{cc} x_{0}+i\cdot \delta_{i}^{\left(c\right)}\cdot \frac{d}{\|d{\|}^{2}}, & \text{for}\;i=1,2,\ldots ,N_{r}^{\left(c\right)}(r\in R_{bg})\\ x_{0}+i\cdot \delta_{i}^{\left(f\right)}\cdot \frac{d}{\|d{\|}^{2}}, & \text{for}\;i=1,2,\ldots ,N_{r}^{\left(f\right)}(r\in R_{buoy}) \end{array}\right.$$

where $\delta^{\left(c\right)}$ is the coarse step size for background sampling, and $\delta^{\left(f\right)}$ is the finer step size for instance‐level sampling. In our experiments, we set up $\delta^{\left(c\right)}\;>\;5*\delta^{\left(f\right)}$ based on our observation of buoy visibility.

In this way, rays are properly classified and routed to train separate NeRF models for their corresponding regions. Additionally, the buoy regions $R_{\text{buoy}}$ are adjusted to sample more densely compared to the background regions $R_{\text{bg}}$. This ensures that fine details of the buoys, such as edges and textures, are accurately preserved while computational resources are efficiently allocated by avoiding unnecessary focus on less critical areas. The adaptive ray sampling technique plays a crucial role in buoy NeRF learning to ensure the visibility of small buoys in the reconstruction, where uniform sparse ray sampling fails to capture them effectively.

### 3.3 NeRF Network and Training

#### 3.3.1 Baseline NeRF Model

To build the NeRF network for density and appearance field estimation, we employ a similar baseline network structure as in the work by Tang et al. (2023). Both density and color are modeled through a multiresolution hash grid encoder paired with shallow MLPs (denoted as E(⋅) for density and H(⋅) for color separately). Features are encoded hierarchically from different resolutions of the hash grid encoder and then are concatenated to input into MLP networks to transform them into density and color values. The density MLP network G(⋅) consists of two hidden layers, each containing 32 hidden channels, to generate a single‐channel density feature. The appearance MLP network P(⋅) consists of three hidden layers with 64 hidden channels to generate a three‐channel color feature. Additionally, we employed appearance decomposition with separate MLPs $P_{d}$ and $P_{s}$ for diffuse and reflective components, respectively. This is particularly suitable for scenarios with reflective water surfaces.

**Volumetric Rendering.** Through the NeRF network, the density of background $\sigma_{c}$ and buoy instance $\sigma_{f}$ are determined as follows:



(5)

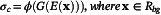

where ϕ is an exponential activation function that promotes sharper surfaces, 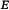 is the learnable multiresolution feature grid for each region's modeling. $x\in R^{3}$ denotes the position of any 3D point sampled from respective regions. The diffuse color and specular color of the water surface are determined as follows:

(6) $$c_{c,d},f_{c,s}=\psi (P_{d}(H_{d}(x))),\mathrm{where}\;x\in R_{bg}$$

(7) $$c_{c,s}=\psi (P_{s}(f_{c,s},d))$$

where ψ denotes the sigmoid activation, and $f_{s}$ represents the intermediate features from the diffuse MLP used for specular color estimation at position x. H(⋅) represents the learnable multiresolution feature grid for diffuse color. Note that the specular color is view‐dependent, so d represents the view direction. Similarly, the modeling of instance‐level color $c_{f,d}$ and $c_{f,s}$ uses the same network structure but is trained with rays $x\in R_{\text{buoy}}$.

Through the volumetric rendering, the final pixel color $\hat{C}(r)$ is calculated by numerical quadrature using the following equation:

(8) $$\hat{C}(r)=\sum_{i}T_{i}\alpha_{i}c_{i},\;\mathrm{where}\;T_{i}=\prod_{j<i}(1-\alpha_{j})$$

with $\alpha_{i}=1-\exp(-\sigma_{i}\delta_{i})$ is the alpha value at point i on the ray that represents the point‐wise rendering weight where $\sigma_{i}$ is the density values. $c_{i}$ is the combined color of diffuse and reflection, where each $c=c_{d}+c_{s}$, and $T_{i}$ is the transmittance, which represents the accumulated product of the (1– $\alpha_{j}$) terms for all j less than i. The rendering loss is used to optimize the baseline model, which minimizes the difference between each pixel's predicted color $\hat{C}(r)$ and the ground truth color C(r)

(9) $$L_{\text{render}}=\sum_{r}\|C(r)-\hat{C}(r){\|}^{2}+\gamma \|c_{s}(r){\|}^{2}.$$

where γ is the weights of specular color regularization.

#### 3.3.2 Multi‐NeRFs with Region‐Specific Extensions

All scene regions, including the mountain, water surface, and buoy instances, are modeled using separate NeRFs. Each model is trained with rays sampled from its designated region of interests segmented in the preprocessing step. This separation is motivated by the distinct characteristics and modeling requirements of each region. For instance, the water surface is dynamic and reflective, with a focus on capturing reflections, lighting variations, and planar structures, while the buoy region is small and spatially distributed, requiring a detailed representation of material properties, shape, and texture. In contrast, the mountain region comprises large homogeneous areas with consistent details. By isolating each region, the feature grids, density, and appearance networks can encode region‐specific characteristics effectively, and further refinements can be applied independently without entangling information from other regions. Additionally, the separation of the buoy NeRF, combined with denser ray sampling, enables the accurate reconstruction of small‐scale buoy instances within a large‐scale background environment.

In our experiments, all models use the same geometry and appearance network architecture for density and appearance field estimation. For the feature grid designed for each region (E(⋅) for density, and H(⋅) for color in the above equations), a consistent resolution (16 levels in our experiments) is applied. Each region's feature grid is aligned with the corresponding physical space. Since the buoy region distributes on the water surface, it shares some spatial extent with the water region. However, feature learning is more focused on the buoy locations, which are controlled by region‐specific masks for denser ray sampling.

After training the separate NeRF models for each region, they are fused together. During inference, rays are sampled from the scene and routed through the appropriate NeRF model based on region‐specific masks. These spatial masks ensure that rays passing through the scene interact only with the relevant feature grid. For instance, rays in the buoy region will interact solely with the buoy feature grid. In regions where the water and buoy areas overlap, the output from the buoy NeRF takes precedence, replacing the overlapping portions of the water mesh. This hard compositing ensures buoy geometry and texture are preserved without mixing with water‐surface geometry.

#### 3.3.3 Water Surface Geometry Regularization

**FIGURE 5:**
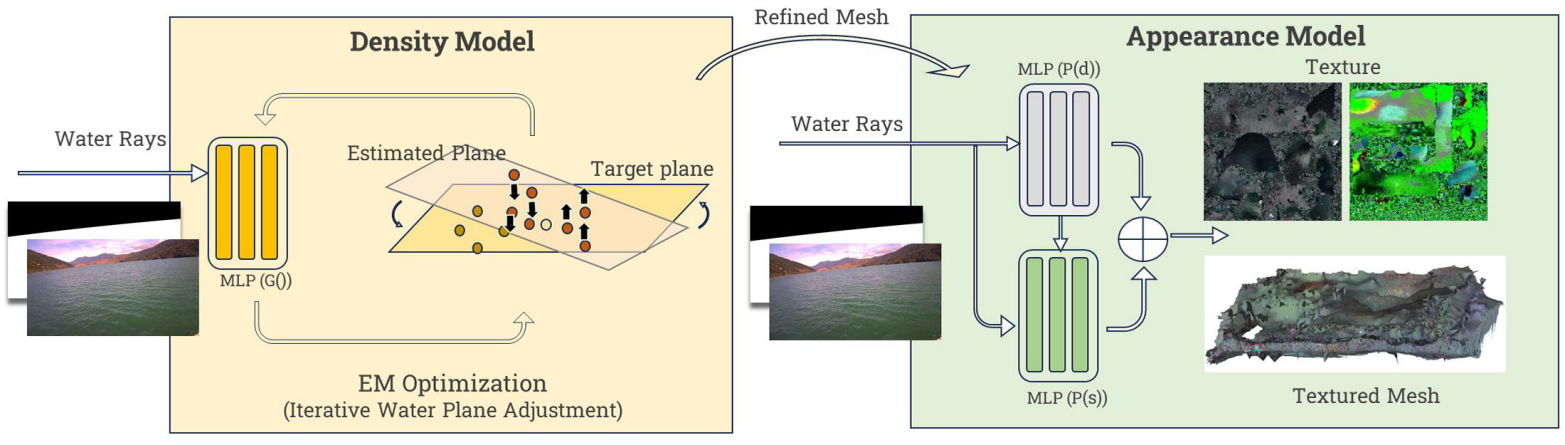
The network structure of water area NeRF for both geometry and appearance modeling.

To improve the baseline model for reconstructing the challenging water surface, we introduce a novel EM algorithm to iteratively perform primary plane alignment during NeRF training. Considering that the water surface is typically flat or nearly flat, we assume that all the estimated high‐density values should be concentrated around this plane. To achieve this, we design a loss function that regularizes the density distribution, prompting the reconstructed 3D points to lie around the flattened area while penalizing outliers that deviate from this primary plane (see Figure 5).

The enhanced NeRF training process is as follows:

**(1) Primary Water Plane Initialization.** To begin, we use the sparse point cloud generated by COLMAP (Schonberger and Frahm 2016) to initialize the primary water plane parameters. The primary water plane $\pi_{0}$ is estimated from this coarse point cloud using the RANSAC method (Fischler and Bolles 1981), leveraging the water region information provided by the mask. The plane parameters, including normal vector n and offset d, are extracted from this initial estimation.

**(2) Expectation Step (E‐step).** Then for each NeRF density sample from one ray, their deviation distance $d_{i}$ to the primary water plane π is computed with the following equation:

(10) $$d_{i}=\frac{\left|n\cdot x_{i}+d\right|}{\left\|n\right\|}$$

where $x_{i}$ is the position of the density sample. We then assign a weight $w_{i}$ to each sample based on its deviation distance from the plane, with samples closer to the plane receiving higher weights and those further away receiving lower weights. This proximity‐based weighting ensures that points near the plane are more influential in the loss calculation.

The weights for ray i are determined using a Gaussian function. The formula for the weights is

(11) $$w(x_{i})=\exp\left(-\frac{\|x_{i}-x_{\text{nearest}}{\|}^{2}}{2k^{2}}\right)$$

where k determines how quickly the weight $w(x_{i})$ decreases as the distance $d_{i}$ from the plane increases, thereby influencing how strongly the regularization term penalizes deviations from the primary plane. For each ray, $w(x_{i})$ center at the data points that are detected closest to the plane (denoted as $x_{\text{nearest}})$ along the whole ray according to their distance $d_{i}$.

**(3) Maximization Step (M‐step).** This step is to update the NeRF parameters to align with the primary water plane. It involves modifying the weights and biases of the MLP layers to ensure that the output density $\sigma_{i}$ conforms to the expected distribution along the primary water plane. The regularization loss function can be represented as

(12) $$L_{\text{plane}}=\sum_{i}w(x_{i})-\sigma_{i}=\sum_{i}\exp\left(-\frac{\|x_{i}-x_{\text{nearest}}{\|}^{2}}{2k^{2}}\right)-\sigma_{i}$$

The total loss function for NeRF training includes the standard NeRF volumetric rendering loss combined with the plane regularization loss:

(13) $$L_{\text{NeRF}}=L_{\text{render}}+\lambda L_{\text{plane}}$$

where λ is a weighting factor controlling the influence of the plane regularization term.[Boxed-text snz270044-alg-0001]

Algorithm 1 **EM Algorithm for Water Surface Regularization**

1 **Input:** Point Cloud *point _loud*, Mask *mask*, Samples *samples,* Epochs *epochs,* Weight Factor *λ***Output:** Optimized NeRF Parameters**Step 1: Water Plane Initialization**Extract water points: *water_point*s ← ***ExtractWaterPoints***(*point_cloud, mask*)Estimate water plane with RANSAC: *normal, offset* ← ***EstimatePlaneRANSAC**(water_points)***Step 2: Expectation Step (E‐step)foreach** *epoch in epochs* **doforeach** *Sample in samples* **do**Compute distance *d* and Gaussian weight: *weight* ← ***ComputeGaussianWeight***(*d*, *k*)Assign weight: *sample.weight* ← *weight***endendStep 3:** **Maximization Step (M‐step)**Initialize loss: *loss_plane ← 0***foreach** *Sample in samples* **do**Update loss: *loss_plane ← loss_plane + (sample.weight − sample.density)***end**Update NeRF parameters with backpropagation.**Step** **4:** **Plane Update***normal, offset* ← ***UpdatePrimaryPlane**(samples)***Repeat Steps 2–4 until convergence.**

**(4) Iterate until Convergence.** After training for several epochs, the estimated water plane is updated. The regularization loss function is then calculated based on the new plane, which is assumed to improve progressively during the training process. This iterative process continues through the E‐step and M‐step until convergence is achieved.

#### 3.3.4 NeRF to Surface Mesh and Texture

Following the NeRF network, we use the Marching Cubes (Lorensen and Cline 1998) method to extract mesh from the NeRF‐estimated density field. Then an iterative mesh refinement is applied to clean the inaccurate vertices and fragmental faces that NeRF may generate, as in Tang et al. (2023). Meanwhile, the NVDiffrast method (Munkberg et al. 2022) is used to perform differentiable rendering and bake the pixel‐wise texture maps.

In summary, compared with existing methods, our approach introduces three key innovations that distinguish it from prior work: (1) a region‐aware scene decomposition tailored to marine environments, which explicitly separates the water plane, background regions, and small buoy instances, in contrast to the spatial tiling or sparsity‐based strategies used in Block‐NeRF (Tancik et al. 2022) and Mega‐NeRF (Turki et al. 2022); (2) an EM‐based water‐plane regularization designed specifically for reflective planar water surfaces, providing a domain‐specific geometric prior that is absent from general large‐scale NeRF frameworks; and (3) an adaptive ray sampling strategy coupled with a dedicated instance‐level NeRF for small and sparse objects such as buoys, enabling the preservation of fine geometric and appearance details.

## 4 Experiments

To evaluate the results, we utilized videos captured from a mussel farm in the Marlborough Sounds of New Zealand, recorded at different times of the day and across various areas of the farm. The footage, captured using a marine camera mounted on a moving vessel, was affected by unpredictable movements, such as hovering and abrupt speed changes, which introduced irregular and inconsistent visual overlaps in the scene data. Additionally, varying illumination conditions in the dataset, including backlighting and highly reflective lighting scenarios, also present further challenges for 3D reconstructions of the mussel farm.

We selected four video clips to evaluate our modules. The details are listed in Table 1. Each video was recorded at 15 frames per second, capturing approximately 20–54 mussel floats at varying distances within each frame. The images extracted from these videos are 1920x1080 pixels in size. We evaluated key stages in the proposed pipeline, including image segmentation for adaptive sampling guidance (Section 4.1), NeRF reconstruction (Section 4.2), and water plane reconstruction, as well as the novel view of the synthesis ability of the model (Section 4.3).

**TABLE 1:** A summary of the video data used for performance evaluations.

Scene	Time length, s	Frames
Scene 1	55	825
Scene 2	51	776
Scene 3	104	1574
Scene 4	67	1008

### 4.1 Waterline and Buoy Detection Performance

Our method relies on precise waterline and buoy detection to identify regions of interest, particularly for nuance recovery. The accuracy of these detection results directly impacts the final reconstruction of each region. A higher number of detected buoys leads to more comprehensive 3D reconstructions. In evaluating both waterline and buoy detection performance, we compared our approach with the state‐of‐the‐art Segment Anything model (Kirillov et al. 2023), which trained on a massive dataset of 11 million images. For the Segment Anything model, we employed the pretrained “vit_h” version of the model, which is available on its official website.

#### 4.1.1 Waterline Detection Performance

Waterline detection is crucial for accurate water surface recovery and refinement in 3D reconstructions. An incorrect water mask can degrade EM plane initialization and regularization, producing less accurate water geometry. We compared the adopted traditional edge detection and filtering method with the Segment Anything approach. In the Segment Anything method, the waterline is identified by the bounding box around the detected water surface, while in the edge detection and filtering method, it is pinpointed as the line at the junction of the mountain and water. To assess performance, we compared the detected line from both methods against the ground truth and calculated the pixel‐wise positional shift along image width using mean absolute error (i.e., L1) and root mean square error (RMSE) metrics.

The comparison of waterline detection methods, as presented in Table 2, shows that the Segment Anything approach consistently outperforms traditional edge detection, yielding lower L1 and RMSE values across all scenes. For example, Scene 4 demonstrates the most significant improvement, with reductions of 0.49 in L1 and 0.81 in RMSE when using the Segment Anything method. This indicates that Segment Anything provides a more precise waterline detection.

**TABLE 2:** Comparison results of waterline detection.

	Edge‐detection		Segment Anything	
	**L1** ↓	**RMSE** ↓	**L1** ↓	**RMSE** ↓
Scene 1	8.25	8.68	7.88	7.93
Scene 2	8.36	8.72	8.01	8.06
Scene 3	8.65	8.85	8.34	8.23
Scene 4	8.02	8.43	7.53	7.62

**FIGURE 6:**
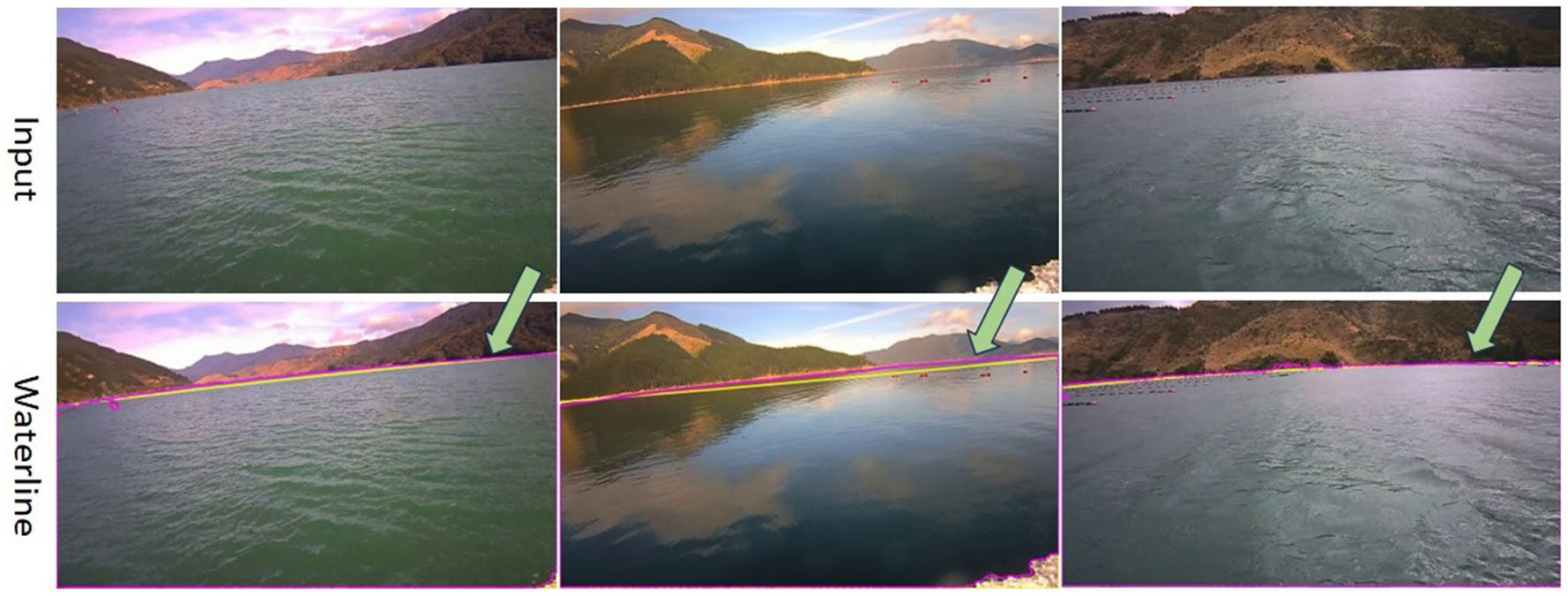
Waterline detection results using the edge‐detection method and the Segment Anything model are shown. In the right columns, the detected waterlines by these two methods are overlaid together on the image for comparison: the red line represents the results from the Segment Anything model, while the yellow line corresponds to the edge‐detection method.

However, while Segment Anything offers a more accurate line approximation, it is more susceptible to interference from surrounding details near the waterline. For instance, as shown in the bottom left row of Figure 6, the waterline becomes curved due to small objects near the detected line, which caused the model to misidentify them as part of the waterline. In contrast, the edge‐detection method, though less precise, produces smoother waterline detection and demonstrates greater robustness across various test data. Considering the usability and stability of the application, we chose the edge detection method in our experiments.

#### 4.1.2 Buoy Detection Performance

**FIGURE 7:**
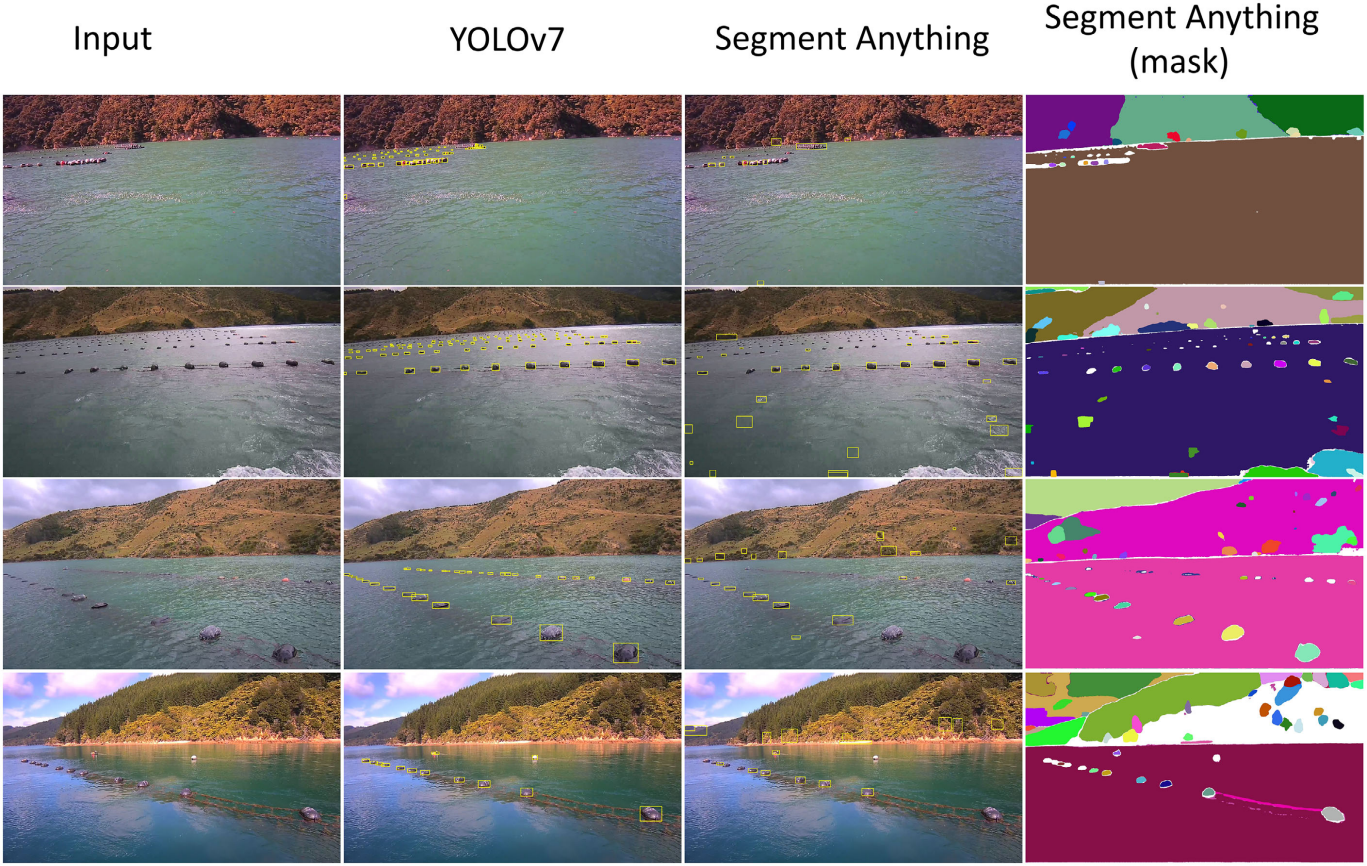
Buoy detection results using the YOLOv7 model and the Segment Anything model are presented, along with the segment masks generated by the Segment Anything model. The comparison shows that the YOLO model we developed performs better on this specific task than the Segment Anything model.

Buoy detection accuracy is critical for precise buoy instance reconstruction. Detection errors affect training, for example, false negatives in buoy detection can cause missed buoy NeRF training leading to absent buoys, while false positives route background rays into buoy NeRF and may introduce spurious small geometry. We first assess buoy detection performance using key metrics: precision, recall, F1 score, and mAP at 0.5 Intersection over Union (IoU). The evaluation was conducted on all frames extracted from four videos, with each image resized to 549x1028 pixels. All the bounding boxes of the detected object located within the water area are evaluated against the labeled ground truth. The qualitative and quantitative results are shown in Table 3 and Figure 7.

**TABLE 3:** Comparison results of buoy detection.

		**Precision** ↑	**Recall** ↑	**F1** ↑	**mAP@0.5IoU** ↑
Scene 1	YOLOv7 (Ours)	0.80	0.51	0.62	0.58
	Segment Anything	0.38	0.11	0.17	0.06
Scene 2	YOLOv7(Ours)	0.79	0.43	0.56	0.45
	Segment Anything	0.33	0.07	0.11	0.03
Scene 3	YOLOv7(Ours)	0.94	0.87	0.87	0.92
	Segment Anything	0.17	0.5	0.26	0.18
Scene 4	YOLOv7(Ours)	0.96	0.86	0.90	0.89
	Segment Anything	0.12	0.68	0.19	0.21

The results reveal that while Segment Anything excels in detecting common objects, it struggles with the specific task of buoy detection in mussel farm environments. In contrast, the retrained YOLOv7 model on mussel farm data demonstrated superior generalization across varying environmental conditions while maintaining a smaller model size and lower computational cost. The YOLOv7 model achieved a peak precision of 0.96, recall of 0.87, F1 score of 0.90, and mAP of 0.92. In comparison, Segment Anything frequently produced false positives in water regions and was often misled by other objects, such as trees in the background, making it difficult to filter out irrelevant segments. Additionally, it struggled with detecting small or distant buoys.

**FIGURE 8:**
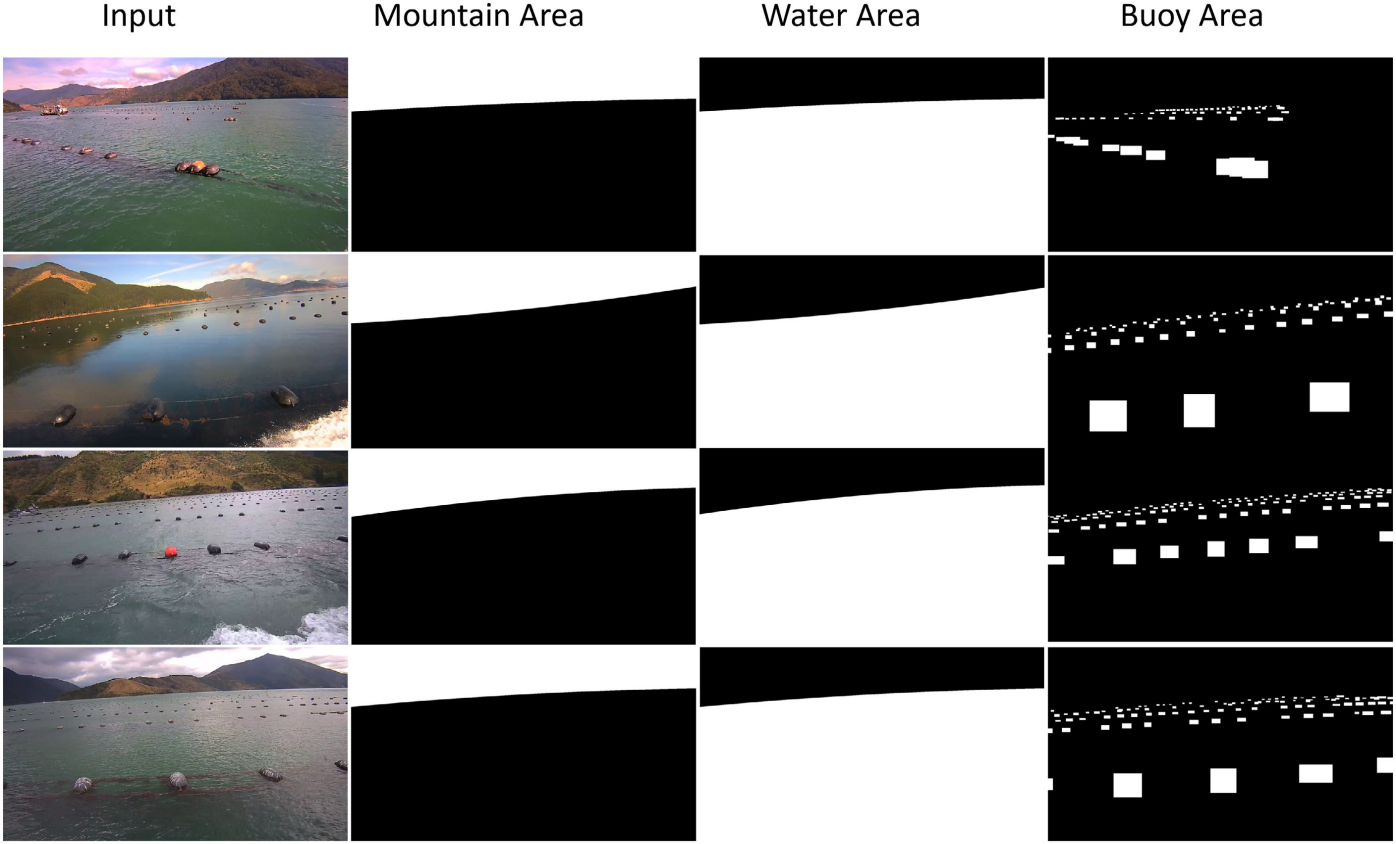
Masks that are used for layer‐wise adaptive ray sampling.

Based on the detection results, masks are generated from the bounding boxes. To ensure the ray sampling for the buoy instance adequately covers all necessary areas for NeRF modeling, we expand the mask of these areas by a factor of 1.1 in our experiment, creating the active buoy area. This also helps tolerate slight localization errors in buoy detection. The generated masks are visualized in Figure 8.

### 4.2 3D Reconstruction Results

In our experiments, the NeRF model training procedure consists of two sequential stages: the density model is trained first, followed by the appearance model, which uses the density model as a geometric foundation. In our experiments, all models are trained on an NVIDIA RTX A6000 GPU in camera‐centered mode without any image downscaling. Given that the mussel farm scene is unbounded, we employ a space warping to map this unbounded space to a bounded one, similar to the approaches used by (Gao et al. 2022; Barron et al. 2022; Zhang et al. 2010; Wang et al. 2023). The learning rate is set to 0.01, and each batch contains 4096 rays. The density model is trained for 160 epochs. To ensure a good initialization, the EM algorithm is applied starting from the 50th epoch and updates the water plane every 5 epochs. The appearance model is trained for 90 epochs. Results are averaged across four test scenes. Training the full Multi‐NeRF pipeline required ≈16 h per scene, including 6 h for background density training with EM, 5 h for background appearance training and mesh refinement, 3 h for buoy density training, and 2 h for buoy appearance training with mesh refinement. In comparison, a baseline single‐NeRF model with the same architecture but without EM or adaptive buoy sampling required approximately 10 h per scene. After training, novel‐view rendering at 1920 × 1080 resolution took about 19 s per frame.

Following the approach by Tang et al. (2023), iterative mesh refinement is performed to enhance the accuracy and detail of both the geometry and appearance of the mesh. Refinement is carried out using a series of ratios set as [0.1, 0.2, 0.3, 0.4, 0.5, 0.7]. The mesh undergoes several optimization iterations where regions with a high error are subdivided to increase detail, while areas with a lower error are decimated to simplify the mesh and reduce computational load. The triangle size threshold is set to 0.01, and the decimation ratio is set to 0.1 in this process.

#### 4.2.1 Comparison with Other Works

**FIGURE 9:**
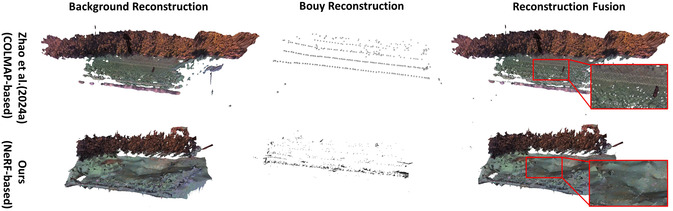
Background and buoy 3D reconstruction results of our method compared to Zhao et al. (2024). It is noteworthy that while sparse ray sampling applied to the background can recover the large‐scale environment, it fails to capture small‐scale buoy instances. By introducing the adaptive ray sampling technique with a focus on the buoy region, combined with buoy‐specific NeRF modeling, effective buoy reconstruction is achieved within the NeRF framework. Compared to Zhao et al. (2024a) triangulation method, which uses a predefined 3D cylinder to represent the buoy model, our approach reconstructs buoys with greater fidelity, accurately preserving their color, shape, and scale. This is particularly important for applications requiring precise modeling of mussel farm environments, although minor background noise is introduced in the process. The water plane recovered by NeRF better reflects the real dynamics and is more complete compared to the COLMAP method, which introduces several holes and reduced area extent due to the loss of correspondence caused by reflections.

We compared our NeRF‐based mussel farm reconstruction method with the approach proposed by Zhao et al. (2024), which is primarily based on COLMAP, an SfM and Multi‐View Stereo pipeline for mussel farm reconstruction. The 3D reconstruction results of the scene background, as shown in Figure 9, demonstrate that our NeRF‐based method improves upon the approach proposed by Zhao et al. (2024), particularly in the completeness of the water area, which is our main area of interest. Zhao et al.'s method, which relies on COLMAP, faces challenges in accurately reconstructing fine details in water areas with complex lighting and reflection conditions. As a result, several holes and a reduced extent of the reconstructed water plane are present in the middle. In contrast, our NeRF‐based method produces more complete and accurate reconstructions of the water area using the same input data. COLMAP/SfM methods rely heavily on reliable local feature correspondences and sufficient parallax; consequently, reflections and low‐texture water surfaces often result in sparse or incomplete point clouds. In contrast, our Multi‐NeRF framework is more tolerant of weak or missing local features due to its volumetric neural rendering formulation, enabling novel‐view synthesis even with fewer reliable correspondences. However, NeRF‐based approaches incur higher GPU training costs and more expensive inference for novel‐view rendering and mesh extraction. By comparison, COLMAP offers faster CPU‐based SfM/MVS pipelines, but typically requires dense feature matching and additional postprocessing for texture baking. In terms of scalability, SfM naturally scales to large image collections, whereas our Multi‐NeRF achieves scalability through region‐wise decomposition, with overall training time increasing as additional region models are introduced.

Moreover, our experiments show that sparse ray sampling of the water surface alone fails to reconstruct the buoys. However, the proposed adaptive ray sampling, along with separate NeRF modeling for the buoy region, effectively enables buoy reconstruction within the NeRF framework (as shown in the middle of the second column in Figure 9), achieving a cohesive representation of both large‐scale environment and small‐scale elements. The adaptive ray sampling technique allows for better capture of subtle variations in instance‐level features. Since this method does not rely on any predefined 3D models for the buoys, as utilized by Zhao et al. (2024), the resulting reconstructions exhibit real boundaries and more precise scales. This improvement is crucial for applications requiring precise modeling of mussel farm environments, as it ensures that critical features are accurately represented and not lost or misrepresented.

Since the NeRF‐based method treats the entire scene within a bounded box, we set the bounding box boundaries to far = 28, near = 0.05, and scale = 0.1. A larger bounding box results in more sampled data points and higher computational costs. To focus the model on the most critical areas, such as water and buoy instances, the NeRF model excludes the more visible mountain regions that extend beyond this bounding box (as shown in Figure 9). While the results in noticeable cutoffs of the mountain area compared to Zhao et al.'s results generated by the COLMAP‐based method, it does not affect the practical application.

#### 4.2.2 3D Reconstruction Ablation Study

**FIGURE 10:**
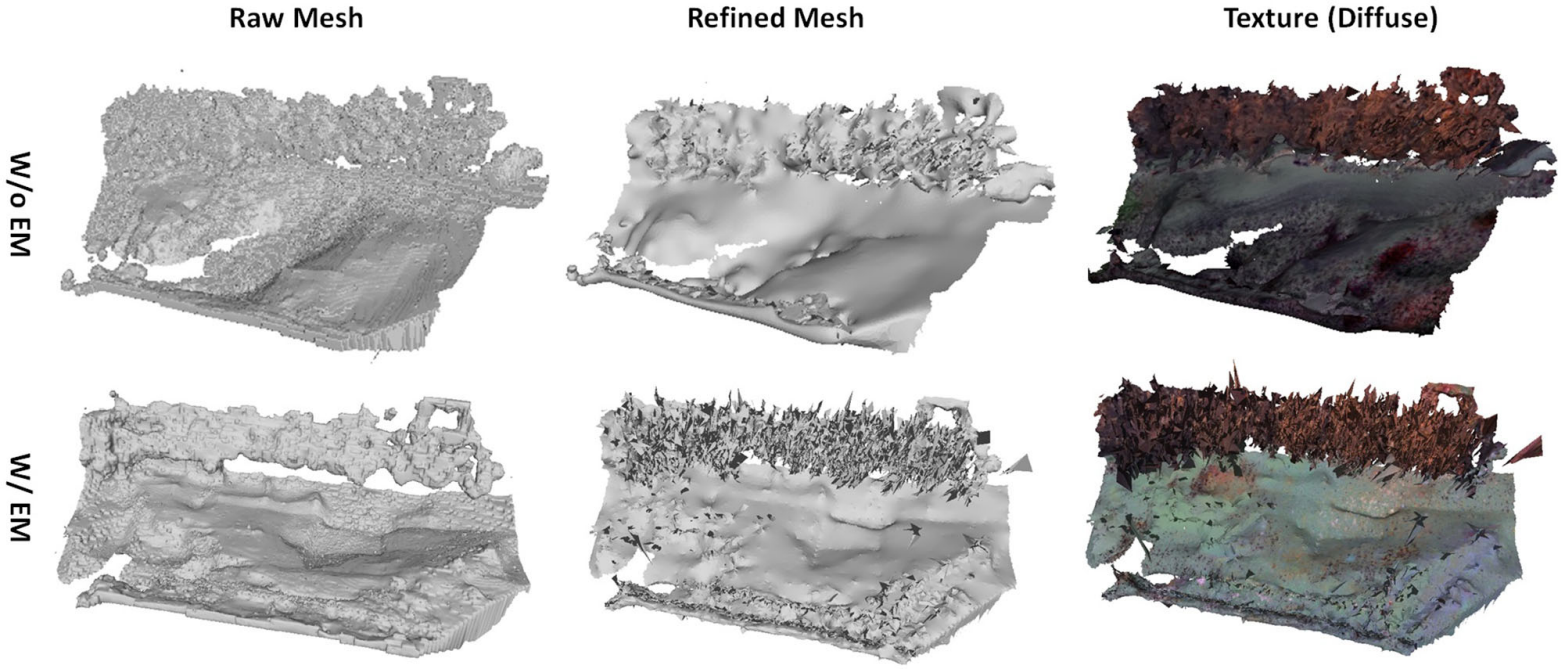
Ablation study on the background reconstruction of the mussel farm scene, comparing the baseline method with the application of adaptive ray sampling and the EM algorithm for water surface refinement. The results illustrate how the proposed methods progressively improve the completeness and accuracy of the water surface reconstruction.

We conducted an ablation study on the background reconstruction performance. We compared our multi‐NeRF‐based method against the EM algorithm for water plane optimization. The results are presented in Figure 10. It demonstrates that the baseline NeRF model alone does not produce competitive reconstruction results (Figure 10, first row), particularly in terms of water surface completeness, compared to the COLMAP‐based method adopted in (Zhao et al. 2024) (Figure 9, first row). However, the addition of the EM algorithm further enhances water surface reconstruction, effectively improving results due to its plane regularization effects (Figure 10), second row). Moreover, the results of the mesh refinement are presented in the second column of Figure 10. From the results, we can see that the refinement process produces smoother meshes. This improvement in mesh quality directly contributes to the overall fidelity of the texture mapping.

#### 4.2.3 Appearance Decomposition: Diffuse and Specular Components

**FIGURE 11:**
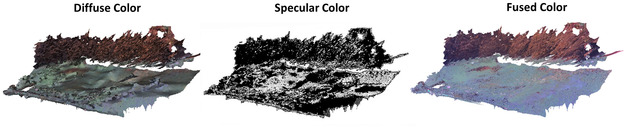
Results of appearance decomposition, including diffuse and specular color components and their fusion.

By separating the appearance network into diffuse and specular MLP ( $P_{d}$ and $P_{s}$) for appearance modeling, we effectively break down the visual aspects into diffuse and specular components as shown in Figure 11. During the appearance model training, we set the specular loss weight γ to $1\times 10^{-5}$ to introduce specular regularization. Since the appearance model is trained based on a well‐trained geometry model, the refined mesh geometry achieved through our method provides a robust foundation for accurately mapping both diffuse and specular properties during appearance recovery. The diffuse component captures the inherent color of the objects, independent of lighting conditions, while the specular component reflects and highlights light interaction. This decomposition enables a more accurate representation of the mussel farm scene, effectively capturing the nuanced interplay between light and surface materials.

### 4.3 Novel View Synthesis

Novel view synthesis allows for the creation of new viewpoints from captured videos. It represents a significant advancement of NeRF techniques beyond traditional 3D reconstruction methods, such as COLMAP. In the context of a mussel farm environment, this technique provides photographic‐like representations of the mussel farm from various angles, which is useful for accurately assessing the farm's layout and conditions without physically inspecting every area. It helps in detecting subtle changes and anomalies that may not be visible from standard views, thereby improving monitoring and management practices.

In the novel view synthesis experiment, we evaluate the performance of our model by synthesizing new views for both the background and buoy instances. For image frames from one video clip of a mussel farm scene, we selected one frame out of every 9 frames as the test data and used the remaining frames for training. The evaluation was calculated using Peak Signal‐to‐Noise Ratio (PSNR) (Huynh‐Thu and Ghanbari 2008), Structural Similarity Index Measure (SSIM) (Wang et al. 2004), and Learned Perceptual Image Patch Similarity (LPIPS) (Zhang et al. 2018) metrics to measure the quality of the synthesized images compared to the ground truth. For PSNR and SSIM, higher values indicate better image quality, while for LPIPS, lower values signify better quality. For the buoy layer, the 3D reconstruction focuses solely on the buoy instance, and the view synthesis generated by the NeRF model is confined to the buoy area within the image. All metrics are calculated based on these regions by comparing them with the detected buoy areas cropped from the image. A PSNR gap between background and buoy synthesis is observed. This difference arises primarily from variations in image region size and signal‐to‐noise ratio (SNR). Background regions consist of large, continuous textures with substantial spatial redundancy and a higher number of training rays, which favor accurate reconstruction of global structure and result in higher PSNR. In contrast, buoys occupy a much smaller image area, provide fewer effective training samples, and are more sensitive to minor misalignments or color and shape inaccuracies, leading to lower PSNR values.

#### 4.3.1 View Synthesis Results

The results, presented in Table 4, showcase the model's capability to generate high‐quality images. The background synthesis achieves a PSNR of 21.881 and an SSIM of 0.603. The buoy synthesis, which addresses more complex and smaller‐scale features, achieves a PSNR of 14.422, an SSIM of 0.866, and an LPIPS of 0.313.

**TABLE 4:** Quantitative evaluation of novel view synthesis results for both background and buoy elements.

	**PSNR** ↑	**SSIM** ↑	**LPIPS** ↓
Background	21.881	0.603	0.546
Buoy	14.422	0.866	0.313

**FIGURE 12:**
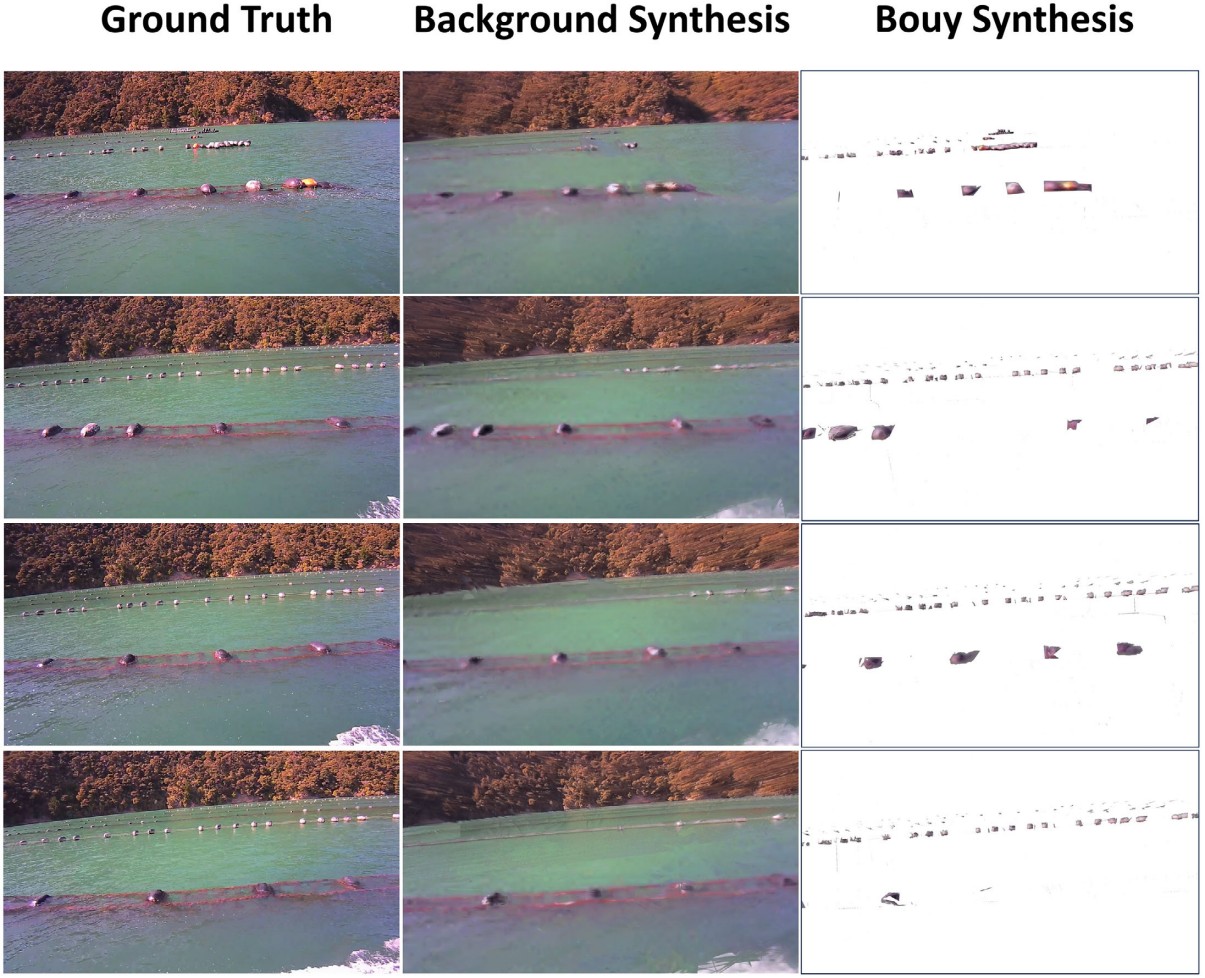
The novel view synthesis of background and buoy instances.

Figure 12 presents the qualitative results of novel view synthesis for both the background and buoy instances. The synthesized views of the background (2nd column) and buoy instances (3rd column) showcase the model's ability to reconstruct the scene accurately from novel perspectives and produce realistic images. This capability stems from the volumetric dense representation of NeRF, which models the scene as a continuous function that can be queried at any 3D point and direction to obtain color and density information. In contrast, the discrete nature of COLMAP's representation, whether through point clouds or meshes, inherently limits the resolution and detail of the synthesized views. When comparing the background view synthesis with the ground truth image, it is noticed that some buoys are missing. However, the buoy NeRF model, which focuses on buoy instances, manages to recover and include such detailed scene information.

#### 4.3.2 Ablation Study on Novel View Synthesis

In addition to the base results, we conducted ablation studies to assess the impact of different components of our approach on novel view synthesis performance. Table 5 presents the quantitative results of these studies. The baseline model achieves a PSNR of 21.372, an SSIM of 0.593, and an LPIPS of 0.560. The inclusion of the EM algorithm further enhances the performance, particularly in terms of PSNR (21.881), SSIM (0.603), and LPIPS (0.546), demonstrating its effectiveness in improving image quality. Finally, the combination of the EM algorithm with buoy adaptive ray sampling yields the best results, achieving a PSNR of 21.932, an SSIM of 0.609, and an LPIPS of 0.538.

**TABLE 5:** Ablation studies on novel view synthesis.

	**PSNR** ↑	**SSIM** ↑	**LPIPS** ↓
Baseline	21.372	0.593	0.560
W/EM	21.881	0.603	0.546
W/EM&Adaptive(Buoy)	21.932	0.609	0.538

**FIGURE 13:**
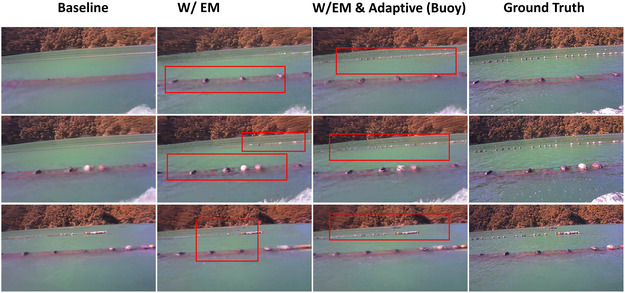
Qualitative ablation studies on novel view synthesis, with each row representing a different example. The results, presented from left to right, demonstrate that the EM algorithm and buoy adaptive ray sampling techniques enhance image quality and progressively reveal additional details.

Figure 13 provides a qualitative comparison of the ablation studies, demonstrating the progressive improvements in image quality as different components are added. The EM algorithm, in particular, enhances the reconstruction of fine details by optimizing the water surface plane. This optimization allows for capturing more detailed information, making additional buoys visible, as demonstrated in the second column of Figure 13 compared to the first column of the baseline view synthesis results. Additionally, incorporating buoy NeRF with adaptive ray sampling on the buoy region further reveals small‐scale details of buoy instances, resulting in more detailed synthetic views, particularly for small buoys located farther from the camera. This is evident in the third column of Figure 13, where overlaying the synthesized buoy on the background image reveals more buoys in the second line, enhancing the overall visibility.

## 5 Conclusions and Future Work

In this study, we present advanced NeRF techniques for novel view synthesis and 3D reconstruction of large‐scale and open environments of mussel farms. By utilizing the NeRF framework and integrating innovations such as the EM algorithm for optimizing water surface reconstruction and buoy adaptive ray sampling for capturing small‐scale details, we have effectively enhanced the quality of reconstruction of mussel farm scenes. The comprehensive quantitative and qualitative results demonstrate that our methods achieved more accurate 3D reconstruction and higher quality of view synthesis compared to baseline models.

Building on the advancements achieved in this study, future work will focus on key areas to further enhance the capabilities of our NeRF model for mussel farm reconstruction. Experiments on the four scenes demonstrate that our method is robust to moderate camera motion and illumination variations, benefiting from volumetric rendering and region‐wise decomposition in NeRF. However, extreme motion or strong directional lighting can still degrade performance and, in severe cases, lead to reconstruction failures. As a next step, we plan to integrate more advanced algorithms and optimization strategies to better handle highly variable environments and to improve robustness under diverse and extreme lighting conditions (Zhao et al. 2021, 2024a, 2024b, 2025). Potential avenues include embedding lighting estimation methods and image delighting techniques to enhance the model's performance. Future research will also include refining the buoy instance synthesis to handle even finer details. By addressing these areas, we seek to advance mussel farming by providing detailed and reliable insights into farm conditions, ultimately supporting better management and optimization of aquaculture practices.

## Funding

This study was supported by Ministry of Business, Innovation and Employment (RTVU1914), Marsden Fund of the New Zealand Government (C11X×2001 and UOCX2104) and Victoria University of Wellington's Strategic Research Cross‐Disciplinary Fund (USRF5101).

## Conflicts of Interest

The authors declare no conflicts of interest.

## Data Availability

Research data are not shared.
